# The intersection between host–pathogen interactions and metabolism during *Vibrio cholerae* infection

**DOI:** 10.1016/j.mib.2023.102421

**Published:** 2024-01-11

**Authors:** Sedelia R Dominguez, Phillip N Doan, Fabian Rivera-Chávez

**Affiliations:** 1 Division of Host-Microbe Systems and Therapeutics, Department of Pediatrics, University of California, San Diego, La Jolla, CA, USA; 2 Department of Molecular Biology, School of Biological Sciences, University of California, San Diego, La Jolla, CA, USA

## Abstract

*Vibrio cholerae* (*V*. *cholerae*), the etiological agent of cholera, uses cholera toxin (CT) to cause severe diarrheal disease. Cholera is still a significant cause of mortality worldwide with about half of all cholera cases and deaths occurring in children under five. Owing to the lack of cost-effective vaccination and poor vaccine efficacy in children, there is a need for alternative preventative and therapeutic strategies. Recent advances in our knowledge of the interplay between CT-induced disease and host–pathogen metabolism have opened the door for investigating how modulation of intestinal metabolism by *V*. *cholerae* during disease impacts host intestinal immunity, the gut microbiota, and pathogen–phage interactions. In this review article, we examine recent progress in our understanding of host–pathogen interactions during *V*. *cholerae* infection and discuss future work deciphering how modulation of gut metabolism during cholera intersects these processes to enable successful fecal–oral transmission of the pathogen.

## Introduction

**Figure 1:**
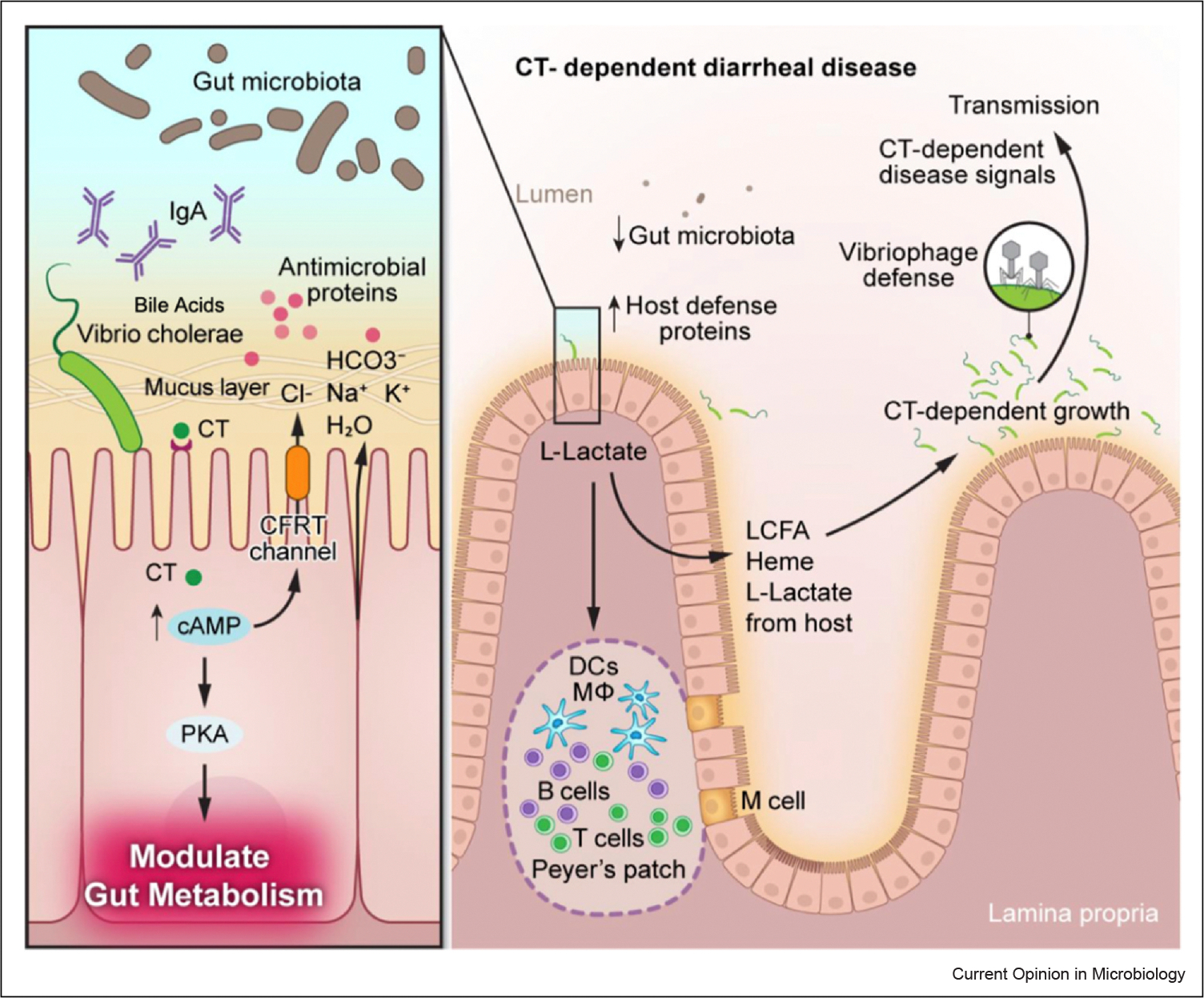
Host–pathogen interactions and metabolism during *Vibrio cholerae* infection. During infection, *Vibrio cholerae* colonizes the ileum of the SI and produces high levels of CT, which activates adenylate cyclase, increasing cellular levels of cAMP. Increased concentrations of cAMP lead to activation of PKA, which phosphorylates and activates the CFTR to secrete chloride ions out of epithelial cells, resulting in an electrolyte imbalance and massive water loss. Increased cAMP and activation of PKA leads to metabolic changes in target cells, which are predicted to lead to the secretion of host-derived LCFAs, L-lactate, and glycerol. During early stages of colonization (left side), *V*. *cholerae* encounters host factors, including bile acids, which act as signaling molecules to activate ToxT-dependent virulence factor expression. *V*. *cholerae* has to overcome components of the innate immune system, including antimicrobial proteins and secretory antibodies, such as IgA, after penetration of the mucus layer. The microbiota in the SI likely imposes ‘colonization resistance’ that *V*. *cholerae* overcomes by unknown mechanisms. The gut microbiota also may compete with *V*. *cholerae* for host-derived nutrients during CT-induced disease that the pathogen overcomes by evolutionary adaptation of the diseased gut as well as its T6SS. CT-induced disease leads to increased secretion of host defense proteins, which the pathogen overcomes by unknown mechanisms. CT-induced modulation of host metabolism promotes the luminal growth of *V*. *cholerae* during infection by acquisition of host-derived nutrients, including LCFAs, L-lactate, and heme. Host-derived L-lactate that is produced during CT-induced disease may play a role in ‘lactylation’ of immune cells in the gut. Furthermore, we propose that CT-induced growth of *V*. *cholerae* plays a role in pathogen susceptibility to phage during infection that the pathogen overcomes by deploying phage defense systems. Altogether, these complex chain events that lead to CT-mediated disease and successful replication of *V*. *cholerae* have been selected for during the evolution of the pathogen in order to ensure fecal–oral transmission of a new host.

*Vibrio cholerae* is the causative agent of cholera, an intestinal bacterial disease characterized by severe diarrhea, vomiting, and hypovolemic shock [1]. Cholera continues to be a significant global health problem causing seasonal and sporadic outbreaks in countries with poor sanitary conditions and limited access to safe drinking water [1]. Despite majority of cases being mild, severe cholera can cause extreme dehydration that results in high incidences of cholera-associated deaths [1]. During infection, *V. cholerae* uses toxin co-regulated pilus to adhere to the small intestinal epithelium and initiate colonization. There, *V. cholerae* secretes an AB5 multimeric toxin, cholera toxin (CT), which can bind directly to intestinal epithelial cells via ganglioside GM1 [1,2]. The catalytic activity of the A subunit (CtxA) activates adenylate cyclase that results in accumulation of 3′,5′-cyclic AMP (cAMP) and downstream cAMP-dependent activation of protein kinase A (PKA). Activation of PKA leads to phosphorylation of cystic fibrosis transmembrane conductance regulator (CFTR), which causes chloride secretion and massive fluid loss out of the intestinal lumen [2]. Secretory diarrhea results from massive fluid loss that is the hallmark symptom of cholera. Our group has recently made the surprising discovery that during infection, CT-induced disease promotes the growth of *V*. *cholerae* in the gut and induces a distinct transcriptome signature in the pathogen that includes the upregulation of a suite of genes involved in virulence and metabolism [3]. Here, we will review the most recent advances in host–pathogen interactions during *V*. *cholerae* infection and discuss how modulation of intestinal metabolism by CT may intercept with processes to drive communicability of disease (Figure 1).

## Modulation of host–pathogen metabolism by cholera toxin

Modulation of host–microbe metabolism by CT has been recently reviewed [2]ece. The severe diarrhea produced during *V. cholerae* infection is thought to be important for the fecal–oral transmission of the pathogen, as up to 10^9^ viable bacteria are excreted per milliliter of stool and remain infectious up to 24 hours after excretion [4]. Recently, it has been found that CT-induced disease promotes the growth of *V*. *cholerae* in the gut by the acquisition of host-derived nutrients, including long-chain fatty acids (LCFAs) and L-lactate [3]. Furthermore, during infection, CT induces a distinct transcriptome signature in the pathogen in both the small intestine (SI) and large intestine. In that study, a suite of *V*. *cholerae* metabolism genes were found to be upregulated in a CT-dependent manner during colonization of the ileum. Interestingly, a unique set of genes were also found to be upregulated in *V*. *cholerae* after it transitions to the large intestine, including genes involved in biofilm formation and chemotaxis [3]. Previous studies have shown that *V*. *cholerae* that is shed during infection is in a ‘hyperinfectious’ state that elevates the fecal–oral transmissibility potential of the pathogen [5]. A more recent study found that growth of *V*. *cholerae* in a biofilm increases hyperinfectivity of the pathogen by the upregulation of virulence genes [6]. Future studies will determine whether CT-induced modulation of host–pathogen metabolism drives fecal–oral pathogen transmission. These studies may point at both host and bacterial metabolism as critical new targets for the development of potential therapies or preventive measures against cholera and other diarrheal diseases.

## Interactions of *Vibrio cholerae* with host immune system in the gut

Cholera is not an inflammatory disease and thus is not associated with a robust recruitment of immune cells during acute infection in humans [7]. This is also observed in animal models of disease where infection with *V. cholerae* and CT-induced diarrheal disease does not elicit a notable immune response in the intestine [8]. However, the mechanisms by which *V*. *cholerae* is able to dampen the host immune response during replication in the gut remain poorly understood. The A and B subunits of CT are known to have anti-inflammatory properties by independent mechanisms [9,10]. Consistent with this, infection with a *V. cholerae* mutant that is unable to produce CT leads to reactogenic diarrhea in both humans and animals [11,12]. Furthermore, *V. cholerae* encodes a multifunctional autoprocessing repeat (MARTX) toxin that has been shown to silence intestinal inflammation and immune cell recruitment [13]. Although infection with *V. cholerae* does not elicit a robust immune response, the innate immune system, including neutrophils, is believed to be important for controlling the pathogen during disease. The recruitment of neutrophils has been observed in cholera patients and shown to be important for controlling *V. cholerae* in animal models of disease [14–17].

In the SI, the intestinal mucus layer serves as a physical barrier against *V. cholerae*. Additionally, secretion of antimicrobial peptides (AMPs) by epithelial cells and production of antibodies in the gut could contribute to defense against *V. cholerae* colonization. It is likely that *V. cholerae* encounters AMPs, including human defensin-5 (HD-5), one of the most abundant in the SI, and plays a crucial role in the defense against pathogens [18]. It has been observed that *V. cholerae* can sense HD-5 and other cationic AMPs *in vitro* [19]. In the presence of AMPs, *V. cholerae* virulence gene expression, including expression of CT, is upregulated, suggesting that sensing of AMPs may contribute to CT-induced host metabolism changes and CT-induced diarrheal disease [19]. A recent study found that quorum sensing signals in *V*. *cholerae* also play a role in the activation of host innate immune responses by repressing pathogen tryptophan uptake and fueling host intestinal serotonin synthesis [20]. As a result of serotonin synthesis, there is increased expression of AMPs, which contributes to increased host cell survival [20]. It is unclear to what extent AMPs impact bacterial colonization and whether CT-induced changes in host and bacterial metabolism genes contribute to AMP secretion. Furthermore, the mechanisms by which *V. cholerae* is able to evade AMP-mediated killing remain unknown. Thus, further assessment of *V*. *cholerae* infection in mice deficient in AMPs may provide further insight into how AMP-mediated *V*. *cholerae* gene regulation contributes to *V. cholerae* metabolism and pathogenesis.

During infection, *V. cholerae* likely encounters Peyer’s patches that are aggregates of lymphoid cells, including B cells, T cells, macrophages, and antigen-sampling dendritic cells. Immunoglobulin A (IgA) is one of the most secreted antibodies by differentiated plasma cells (PCs). PCs can respond to luminal cues and intestinal metabolism to modulate IgA secretion. Notably, dietary cholesterol absorption and production of oxysterols contribute to IgA production [21,22]. 25-hydoxycholesterol (25-HC), an oxysterol metabolite generated by cholesterol 25-hydoxylase (CH25H), is necessary for antigen-specific IgA responses [21]. In a CT-treated mouse model, mice lacking CH25H showed increased CT-specific IgA, suggesting that CH25H and production of 25-HC controls antigen-specific IgA responses [21]. CT modulates intestinal metabolism that can alter downstream metabolic cues necessary for antigen-specific IgA responses. However, whether alterations in gut metabolism by CT play a role in IgA secretion remains unexplored. In addition to luminal metabolism-mediated modulation of IgA, nongenetic transfer of IgA through the entero-mammary axis can influence IgA responses in the gut [23]. Therefore, future studies should examine how metabolite-induced IgA secretion and entero-mammary transfer of antigen-specific IgA impacts CT-induced disease.

High-fat diets (HFD) are associated with downregulation of defensins, and downregulation of peroxisome proliferator-activated receptor (PPAR-y), a nuclear receptor that is involved in lipid metabolism and mucosal defense regulation [24]. HFD can also induce fatty acid-binding proteins (FABP), which have been shown to be involved in downregulation of defensins through PPAR-y degradation [25]. A recent study found that activation of PPAR-y in the SI by a *Lactobacillus*-derived metabolite, phenyllactic acid, alters lipid metabolism in the ileum of the SI [24]. CT is predicted to alter lipid metabolism in the ileum [3]. However, whether PPAR-y signaling, and downstream targets, are altered during CT-induced disease remains unknown. Additionally, studies should investigate whether modulation of PPAR-y-mediated lipid metabolism and mucosal defense regulation impacts *V. cholerae* colonization.

*V. cholerae*-secreted proteases have been identified in infected animal and human stool and may be involved in *V. cholerae* evasion of host innate immunity [26]. Recently, intelectin, which is involved in the clearance of pathogens, was found to bind *V. cholerae in vitro* [26,27]. *V. cholerae* proteases target intelectin for degradation, suggesting that host *V. cholerae* interactions may be modulated by pathogen-secreted enzymes [27]. Analysis of cecal fluid from WT *V. cholerae* and CT-mutant-infected rabbits demonstrated that CT induces secretion of host defense proteins [28]. Surfactant protein D (SP-D), a host lectin involved in innate immunity, was also found to bind *V. cholerae* in a CT-dependent manner and alter *V. cholerae* virulence gene expression [28]. However, the mechanisms by which CT-induced disease leads to increased host defense factors including SP-D and whether this depends on modulation of intestinal metabolism remains unknown. In the absence of proteases, SP-D remained bound to *V. cholerae* for longer, suggesting that proteases may prevent host defense factors from targeting *V. cholerae* [28]. Future studies should characterize secreted proteases and their targets to identify unexplored *V. cholerae* interactions. Additionally, several identified *V. cholerae-*secreted proteases are secreted by the type-II secretion system (T2SS), whose role in *V. cholerae* host–microbe interactions in the gut remains poorly understood [29,30]. Thus, future studies should explore how T2SS-secreted factors, including CT, act in concert during *V. cholerae* infection to modulate host responses, gut metabolism, and bacterial pathogenesis.

Infection with *V. cholerae* leads to a CT-dependent switch in anaerobic glycolysis and increased concentrations of L-lactate in the SI of infected animals [3,31]. *V. cholerae* genetically encodes the ability to take up and utilize L-lactate, and these genes are upregulated in a CT-dependent manner during infection [3]. Recent studies have found that increased lactate concentrations can lead to a reprogramming of immune cells to mediate immunosuppression through lactate-induced histone modification, termed ‘lactylation’ [32,33]. It is possible that immunosuppression by CT is in part due to CT-induced modulation of epithelial cell metabolism leading to lactate-mediated lactylation of immune cells in the gut.

## How the gut microbiota in the small intestine impacts V. cholerae infection

*V. cholerae* survival in the intestinal tract is impacted by the gastrointestinal environment. *V. cholerae* encounters bile acids, changes in pH, and limited oxygen availability, all which the bacterium must overcome to promote its colonization. *V*. *cholerae* also encounters commensal microbes that can impact disease susceptibility. Commensal microbes encoding bile salt hydrolases (BSH) can convert taurine-conjugated taurocholate (TC) and glycine-conjugated (glycocholate) forms of bile into cholate/cholic acid (CA). Previous studies have shown that bile salts, specifically TC, the predominant bile molecule in humans and mice, can induce the expression of *V. cholerae* virulence genes [34,35]. Recently, it was reported that a gut commensal, *Blautia obeum*, can suppress *V. cholerae* virulence by BSH-mediated degradation of TC to CA [36]. Interestingly, humans with cholera have decreased concentrations of *Blautia obeum* and other obligate anaerobes compared with healthy individuals or individuals recovering from the disease [37]. *V. cholerae* can also employ its antibacterial type-VI secretion system (T6SS) to directly kill members of the microbiota [38,39]. Recently, it was discovered that CT-induced disease can lead to increased concentrations of host-derived nutrients [3], that likely also play a role in altering the small intestinal microbiota during *V. cholerae* infection. However, because *V. cholerae* is a human-specific pathogen, microbiota studies have been limited to the use of neonatal animals that have an underdeveloped microbiota (and immune system). Remarkably, a recent study found that targeted depletion of obligate anaerobes, specifically members of the phylum *Bacteroidetes*, leads to susceptibility of adult mice to colonization upon oral challenge by *V. cholerae* through the modulation of microbiota-derived short-chain fatty acids [40]. Thus, this study highlights the role of microbiota-derived metabolites in the susceptibility to *V. cholerae* and opens the door to mechanistic characterization of the role of the gut microbiota and the host immune system during cholera.

## Antiviral immunity implications in the context of cholera toxin disease

*V*. *cholerae* inhabits warm estuaries and is in constant conflict with bacteriophages in both the aquatic and intestinal environments [41]. Recently, a plethora of studies have revealed a fascinating look at the arms race between *Vibrio* species and their phages. Bacteriophage therapy is a promising strategy for combating *V*. *cholerae* infection. A study found that a cocktail of bacteriophages prevented *V*. *cholerae* infection in an animal model of disease [42]. However, some *V*. *cholerae* strains can employ a CRISPR/Cas system and secrete outer membrane vesicles as a defense mechanism against phage predation [43,44]. *V*. *cholerae* also uses a bacterial cyclic oligonucleotide-based antiphage signaling system (CBASS), which provides the pathogen with antiviral immune protection [45]. The CBASS operon encodes a cGAS/DncV-like nucleotidyltransferase (CD-NTase) enzyme that synthesizes a nucleotide second messenger in response to viral infection in which an associated Cap effector protein then binds the nucleotide signal and executes cell death to destroy the host cell and block phage propagation [46]. During infection, CT-induced disease promotes the explosive growth of *V*. *cholerae* in the gut [3]. Thus, *V*. *cholerae* is likely highly susceptible to phage attack during CT-dependent replication in the intestine. It is tempting to speculate that some phage defense mechanisms may have evolved regulatory mechanisms that allow them to be employed during CT-dependent diarrheal disease. *V*. *cholerae* encodes several toxin–antitoxin (TA) systems that are encoded in the ‘superintegron’ of the small chromosome [47]. Recently, it has been proposed that TA systems may also serve as phage defense elements [48]. Interestingly, expression of the small chromosome is amplified in a CT-dependent manner, which may be important for phage defense during CT-dependent growth in the gut [3]. A better understanding of the evolutionary arms race between phages and *V*. *cholerae* antiphage defense mechanisms would be important for the future design of phage therapy to combat cholera.

## Concluding remarks and remaining questions

Although the causative agent of cholera, *V*. *cholerae*, was identified over 140 years ago, we are still learning how this human pathogen causes disease. Recent advances in our understanding of how the pathogen’s central virulence factor, CT, acts as a multifunctional toxin that couples severe diarrheal disease with modulation of gut metabolism represent a paradigm shift in our understanding of *V*. *cholerae*. These recent findings open the door into investigating the interplay between modulation of gut metabolism by CT, host intestinal immunity, the gut microbiota, as well as phage–pathogen interactions during disease. It is important to note that strain-level differences within pathogenic *V*. *cholerae* exist and not all strains encode identical virulence factors and antiphage systems [49]. However, all pathogenic *V*. *cholerae* strains cause disease by utilizing CT which likely represents an important evolutionary determinant in all pathogenic strains. Further understanding of the molecular mechanisms by which CT-induced modulation of gut metabolism drives the growth and fecal–oral transmission of the pathogen will be key for the future design of therapeutic strategies that target both host and bacterial metabolic pathways.

## Data Availability

No data were used for the research described in the article.
